# Automating checks of plan check automation

**DOI:** 10.1120/jacmp.v15i4.4889

**Published:** 2014-07-08

**Authors:** Tarek Halabi, Hsiao‐Ming Lu

**Affiliations:** ^1^ Radiation Oncology Massachusetts General Hospital, Harvard Medical School Boston MA

**Keywords:** plan check automation, chart review, quality assurance

## Abstract

While a few physicists have designed new plan check automation solutions for their clinics, fewer, if any, managed to adapt existing solutions. As complex and varied as the systems they check, these programs must gain the full confidence of those who would run them on countless patient plans. The present automation effort, planCheck, therefore focuses on versatility and ease of implementation and verification. To demonstrate this, we apply planCheck to proton gantry, stereotactic proton gantry, stereotactic proton fixed beam (STAR), and IMRT treatments.

PACS numbers: 87.55.km, 87.55.Qr, 87.55.T‐, 89.20.Bp

## I. INTRODUCTION

Efforts to automate plan checks began even before electronic charts were utilized.^(1)^ Siochi et al.^(2)^ presented a more complex solution for electronic charts, involving several modules written in MATLAB (including CERR^(3)^), visual basic.net, and visual basic 6.0. The need for a meta‐check (a check of a check) was already clear to these authors, who had one of their modules, called “RTP‐filter”, check the work of the other, “LEX”. Another, even larger scale automation effort followed in Yang et al.^4^, ^5^

In spite of these efforts, highly trained and educated physicists of most departments continue to spend large amounts of time manually cross‐checking numbers between planning and record and verify (R&V) systems. At our institution, physicists cross‐compare a plan PDF, uploaded into MOSAIQ R&V, against the latter and against an in‐house treatment planning data base (TPDB). Manual checks prevail because considerable upfront effort is required to build, adapt, and test automations for specific combinations of continually evolving platforms and modalities.

The present automation effort addresses this difficulty by placing much more emphasis on versatility and ease of implementation and verification. Our solution, planCheck, is a universal framework for organizing, acquiring, checking, and displaying data, as well as for verifying integrity of checks. Versatility of plan data acquisition is afforded by a robust PDF parsing facility, given that all planning systems output PDF reports. Versatility and strength of software verification is afforded by a universal facility for failure mode analysis. With over 11 different types of radiation treatments, six planning systems, four vendors, and proton therapy technology that spans multiple eras, the workflow environment at our institution is as good as any to test planCheck's versatility. We currently use it for initial, weekly, and final checks of four treatment types that include both photon and proton modalities.

## II. MATERIALS AND METHODS

### A. Universal framework

#### A.1 Classes

PlanCheck is written in visual basic.net. Good programming practices alone go a long way towards ensuring its ease of implementation and adaptation. By creating a hierarchy of patient classes — general, proton, photon, gantry, fixed beam, stereotactic, etc. — we avoid code repetition through inheritance and restrict properties and methods. These restrictions appear in the design process through IntelliSense (IntelliSense, Woburn, MA), an early debugging program that prevents the exponentially more time‐consuming usual form of later debugging.

To further avoid code repetition, we consolidate as much code as possible into class definitions, as opposed to object instantiations of these classes. Consider, for example, our “param” class, instantiated for each parameter as a member of either a patient class or one of its beams. In its “source” properties, param stores potentially different values obtained from different sources (e.g., plan PDF, RadCalc PDF, R&V database). Code for cross‐comparison of these values and for issuing warnings when mismatches are detected is stored in the param class definition itself, so that it is not repeated for each of its instantiations. We refer to such cross‐comparisons as implicit checks (more on this later) to distinguish them from the more customizable checks made explicit by checkboxes (both automatic and manual). The automatic explicit checks are typically more complex and involve more than simple cross‐comparison. Nonetheless, code for much of their work (e.g., un‐checking boxes, issuing warnings) is, again, not repeated for each check, and is placed in a myCheckbox class definition, derived from Microsoft's checkbox control.

#### A.2 Display

**Figure 1:**
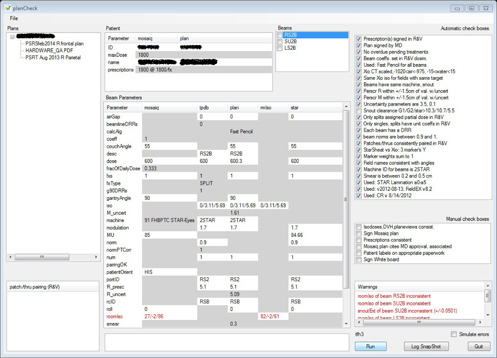
planCheck results for a stereotactic proton fixed beam (STAR) plan.

Given a patient's unit number, planCheck lists in its top left panel (see Fig. 1) the newest versions of all nonvoid plan PDFs that were uploaded for this patient into the R&V. The user then selects a plan and clicks “run”. planCheck then parses the plan, identifies and parses RadCalc PDFs (Life‐Line Software, Inc. Tyler, TX) containing beams with IDs matching those in the plan PDF, collects relevant data from R&V and in‐house treatment planning database (TPDB), checks the data, and displays results.

Patient param values from different sources are tabulated in the Patient panel (See Fig. 1). When declared as a beam property, a param's values are tabulated in the Beam Parameters panel, which updates whenever the user highlights a different beam from the Beams panel. planCheck always cross‐compares values of the same parameter (same row) from different sources (different columns) if they appear on white background. We refer to these automatic comparisons as implicit checks which, unlike explicit checks, are not associated with checkboxes (see Fig. 1). When an implicit check detects an error, the corresponding row in the table turns red and a warning is issued and displayed in the Warnings panel, specifying the error and the offending beam. Implicit checks greatly reduce the number of checkboxes needed.

### B. Plan data acquisition

The goal of a plan check (initial, continuing, or final) is to ensure that the radiation oncologist's intentions, officially recorded in the signed plan document and R&V prescriptions, match what is to be (or has been) delivered. The plan document is typically a PDF, and is assumed here to have been uploaded into the R&V. (Many R&V users, typically those using Aria, do not make use of this PDF upload feature, though it is a feature that is always available to them). It is ultimately this PDF, not any DICOM RT file, that must be checked by the physicist against what is to be delivered; oncologists do not review or sign DICOM RT files.

While this PDF may lack the universal structure afforded by the DICOM RT standard, this is a blessing in disguise. Detailed knowledge of the DICOM RT standard for the many types of constantly evolving treatments at our institution and of the many systems that output them is not required for the present effort. We need only develop a single set of PDF parsing tools that can then be applied to any document from any system (planning, RadCalc, etc.) for any type of treatment. These tools notably include a powerful and easy‐to‐learn language for robust parsing called Regular Expressions.^(6)^ To adapt planCheck to a new system (whether a planning system or a second check system like RadCalc) we would simply create a new module for parsing data from this system's PDF output. Given the limited number of radiotherapy systems on the market, we estimate that planCheck will include parsing modules for all major systems by next year. Users will then not have to worry about this step.

All that is required to run planCheck is the username and password to the R&V database, the IP address to this database, and the path to the data folder containing uploaded PDFs. The connection established with the R&V database is read‐only. However we are aware of the possibility of infinite loop readouts crashing the R&V. We have two measures protecting against such a problem. First, upgrades involving the R&V readout are always run first on a test database that is a backup copy of the clinical R&V database. Second, these upgrades are performed during after‐hours.

### C. Verification

One of the main objectives of verification is to ensure that planCheck does indeed catch the errors it claims to catch. It is important to realize that in‐field testing alone, however, would not be sufficient, since these errors arise at very low frequencies in the clinic. Verification, instead, requires failure mode analysis and self‐testing, as implemented below.

planCheck always channels collected data through text files or dataTables (vb.net) before running its checks. If the user checks the “Simulate errors” checkbox (see Fig. 1) before a run, planCheck displays the content of these text files and dataTables for the user to edit before any of the checks are carried out. When all displayed files and tables have been closed, planCheck runs its checks. If the fake errors trigger the appropriate warnings, unchecked checkboxes, and red colored rows, then (without requiring detailed knowledge of how it operates) the user knows that planCheck functions as advertised. For all treatment types and databases, the same code generates the appropriate dataTable scheme (based on treatment type), displays it, and writes it into the appropriate source property of param objects.

While the above facility allows verification of the algorithms underlying planCheck's automatic checks, it does not verify that the data inputted to these algorithms were correctly read. At our institution, additional tests are therefore performed, particularly after upgrades. These tests and their results are recorded in a log file and, for major updates, are also summarized in a test report file. Although these additional tests involve direct cross‐comparison of data tabulated by planCheck against values displayed by original data sources, it is important to note that readout bugs are almost never discovered by such direct cross‐comparison. They are instead exposed by the false warnings they lead to. This will be the case unless the bug in reading a particular parameter from a particular data source also exists for all other data sources. Such bug conspiracies are fortunately very rare. A main goal of running these additional tests is to expose planCheck to a large variety of plans, while looking for false warnings and for larger scale bugs. For example, we look to make sure that planCheck identified the correct treatment type, that it did not miss any pages from the plan PDF, miss any beams, rows, checkboxes or data sources (columns in the table displays). This is often done by comparing displays to those of the earlier version.

## III. RESULTS & DISCUSSION

**Figure 2:**
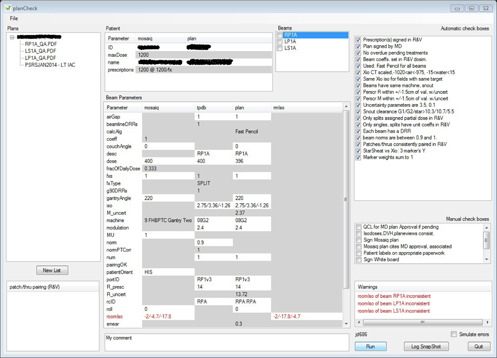
planCheck results for a stereotactic proton gantry plan.

**Figure 3:**
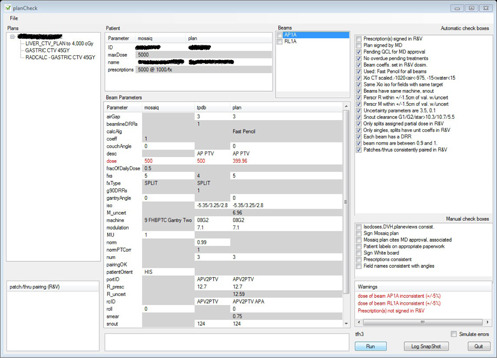
planCheck results for a proton gantry plan.

**Figure 4:**
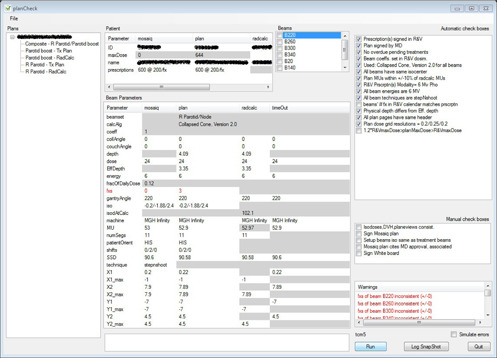
planCheck results for an IMRT plan.

A set of explicit checks is defined for each treatment type. Figure 1 displays planCheck results for a stereotactic proton fixed beam (STAR)^(7)^ plan. The first five automatic check boxes were inherited from the general patient class and appear for all plan types currently handled by planCheck (see also Figs. 2, 3, and 4). Note, though, that the appropriate dose calculation algorithm used by the fifth checkbox is plan type dependent. The first automatic checkbox queries the MOSAIQ database (IMPAC Medical Systems, Sunnyvale, CA) to determine whether prescriptions to which the plan's beams belong have been signed. Similarly, the second checkbox determines whether the radiation oncologist has electronically approved the plan PDF in MOSAIQ by querying the MOSAIQ database. The third checkbox queries the MOSAIQ database looking for pending overdue treatments. The fourth checkbox determines whether the planner has clicked the “Dosimetry” button in MOSAIQ to populate dose coefficients for beam‐region combinations, again by querying the MOSAIQ database. For each treatment type, an appropriate dose calculation algorithm is defined. The fifth checkbox determines whether the algorithm specified in the plan PDF is the appropriate one.

The next 12 automatic checkboxes in Fig. 1 are inherited from the proton XiO patient class (Elekta CMS, St. Louis, MO) (from which STAR patients derive). They, therefore, also appear for gantry proton plans (see Fig. 3) and stereotactic gantry proton plans (see Fig. 2). The first of these checkboxes determines, from the plan PDF, whether the planner has calibrated the CT images and whether the calibration results were reasonable. This process is specific to proton plans and is carried out in the proton XiO planning system.

In XiO, a target structure is defined for each beam. The next checkbox determines, from the plan PDF, whether beams with the same target have the same isocenter. Note that there are legitimate exceptions to this rule, such as when isocenters for abutting beams are shifted at different “levels” of treatment to reduce overlap dose.

Depending on field size, different snout sizes are used in proton nozzles. Snout changes are time consuming, and should be avoided within the same treatment session when possible. The next checkbox determines, from plan PDF, whether all beams in the plan have the same machine and snout size.

When uncertainty margins are included, the range and modulation may differ from prescribed values. The next two checkboxes determine whether the difference is reasonable. The current 1.5 cm threshold on these two checks is admittedly too large, though we are awaiting recommendations from another physicist at our institution on how to refine these two checks.

The next checkbox determines, from the plan PDF, whether some parameters specific to the proton XiO algorithm were set to default values.

Proton snouts extend along the beam axis. In order to avoid collision, these extensions must not exceed machine dependent values. The next checkbox ensures this is the case, again using the plan PDF. Note that this check failed for the plan results displayed in Fig. 1. planCheck automatically unchecked this box, and displayed a warning in the Warnings panel, specifying the offense and the offending beam. This specific warning is not displayed in the figure (one would see it by scrolling down further in the Warnings panel).

When the full daily dose to a target is delivered by a single proton beam that is neither a “patch”, a “thru”, nor an “abutting” beam, this beam is called “single”; otherwise, it is called a “split”. The next two checkboxes determine whether doses and coefficients stored for the plan's beams in the MOSAIQ database make sense based on beam type.

The in‐house database, TPDB, is necessary for proton planning, since it facilitates data transfer to the machine shop that fabricates apertures and range compensators. It also stores (meta‐)data on DRRs. Plan data is exported by the planners into TPDB. The next checkbox determines, by querying TPDB, whether at least one DRR was exported for each beam.

The next checkbox determines, again by querying TPDB, whether the dose scaling for each beam is reasonable. The per‐beam normalization process at our institution is specific to proton beams.

In contrast to photon treatments, the same proton daily dose is often delivered by different beam combinations on different treatment fractions. Nevertheless, patch‐thru beam pairs must always be scheduled together. In other words, if the planner scheduled a patch beam for Tuesday's treatment, he/she should also have scheduled the “thru” beam of the pair on Tuesday. The next checkbox determines whether these beams always appear in pairs in the treatment schedule stored in the MOSAIQ database and that the pairing is consistent. This check would be very time‐consuming and tedious if performed manually. Parings confirmed by planCheck are displayed in the bottom left panel of Fig 1. Note that the plan checked in Fig. 1 did not include any patch/thru pairs.

A fiducial for stereotactic plans is typically digitized on up to three CT slices and the final coordinates are obtained through a weighting procedure. The next two checkboxes confirm consistency of the process, using the plan PDF. These two checks are specific to stereotactic plans and appear for the treatment types displayed by Figs. 2 and 3. An additional report sheet is generated for stereotactic plans, and is designated by planCheck as a data source in and of its own, called rmIso (see columns of Beam parameters panels of Figs. 1 and 2). It stores isocenter coordinates transformed to the treatment room frame of reference, a transformation needed for all proton stereotactic plans. Note that the implicit (across‐row) check of these coordinates failed (see red row in the Beam parameters panels of Fig. 1) because the planner erroneously transcribed these transformed coordinates into MOSAIQ. Such transcription errors are common to proton planning because R&V systems have not yet been properly developed to allow automated import of plan information.

The rest of the automatic checkboxes shown in Fig. 1 are specific to STAR plans. The simple beam naming convention used for these plans allows planCheck to confirm, in the first of these checkboxes, that the beam orientation matches what is described by the name.

There is only one machine used for STAR treatments, and the next checkbox ensures that this machine was chosen for these plan types.

Range compensators in proton therapy are smeared to improve robustness. This smearing extent always falls within a known range for STAR treatments, and the next checkbox confirms this, again using the plan PDF. The final three checkboxes of Fig. 1 confirm, using the plan PDF, that correct versions of various algorithms needed for STAR plans were used. An additional report sheet is generated for STAR plans, and is designated by planCheck as a data source in and of its own, called “star” (see columns of Beam parameters panels of Fig. 1).

Since we only use 6 MV beam energy for IMRT plans at our institution, we've included automatic checkboxes (8th and 9th from top in Fig. 4) confirming that both the individual beam energies, as well as the modality chosen in the MOSAIQ prescription, is 6 MV; similarly for the step‐and‐shoot technique.

For IMRT plans, we've also included an automatic checkbox (11th from top in Fig. 4) that queries the MOSAIQ database to confirm that the number of scheduled fractions for all plan beams matches the one specified in the MOSAIQ prescription to which they belong. Note that such a check would not apply for proton plans, since different proton beams belonging to the same prescription may be treated for different numbers of fractions. Note that this automatic check failed for the plan results displayed in Fig. 4 because the planner had not yet set up the treatment schedule in MOSAIQ. The warning corresponding to this failure is not shown in the figure, but can be seen if one scrolls further down in the warnings panel.

Similarly for the final automatic checkbox in Fig. 4, which also failed. This checkbox confirms that the maximum dose reported in the plan PDF is within 1‐1.2 times the maximum dose “carried” by any MOSAIQ “region” for the plan's beams. A “region” could either be a prescription site or a cumulative dose column created by the planner in MOSAIQ. This check affords valuable immunity against differing conventions for representing concomitant dose plans in MOSAIQ. It failed in this chase because the planner had not yet set up the treatment schedule in MOSAIQ.

The third automatic checkbox in Fig. 3 appears only when the plan PDF has not been signed. This checkbox is common to all plans currently handled by planCheck. It queries the MOSAIQ database to confirm that the planner has prompted the radiation oncologist, through MOSAIQ's QCL facility, to electronically approve the plan PDF. Only when both the second and third automatic checkboxes shown in Fig. 3 fail, is a warning displayed in the Warnings panel.

This leniency can be explained by the additional requirement at our institution that radiation oncologists initially sign the plan PDF vicariously through the planner who notes their approval in the plan PDF. The physicists must check that such a note was left in the plan PDF. This is an example of a manual checkbox listed in planCheck's manual check boxes panel as a reminder for physicists checking the plan.

## IV. CONCLUSION

planCheck does not completely replace a human plan check. The human checker still needs to manually carry out the checks listed (for his or her convenience) in the manual checkboxes panel of planCheck's display. Human checkers are also encouraged to occasionally use the failure mode analysis facility (the check of the check). This is only automated to an extent, of course. The user still has to check the “simulate errors” checkbox, edit in errors, and examine the results.

Future efforts may involve design of quantitative methods to evaluate efficacy, but care must be taken to avoid proving the obvious. Are we proving that a human checker may forget to check a certain aspect of the plan? We all agree that they will do so in the limit of infinite plan checks. Are we proving that the errors caught by planCheck actually occur in the clinic? In our experience with planCheck, this is certainly the case. In fact some of the checks were designed with historic precedence in mind, and the reader can judge the clinical relevance of included checks (which are highly customizable) based on his/her experience.

## Supporting information

Supplementary Material
