# Supplementary figures and images for: Automating checks of plan check automation
Source: J Appl Clin Med Phys. 2014 Jul 8;15(4):392–9. doi: 10.1120/jacmp.v15i4.4889 (PMC7678958; doi:10.1120/jacmp.v15i4.4889)

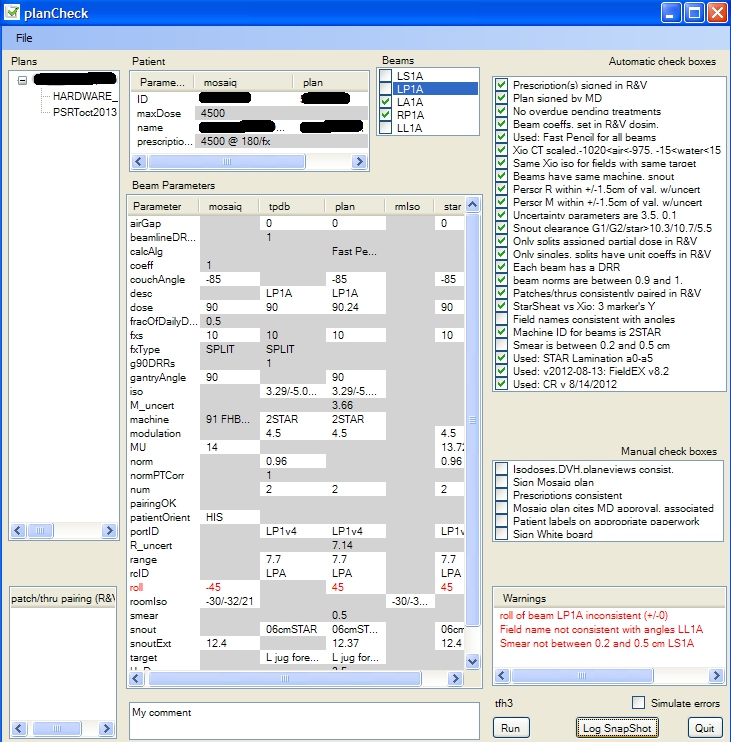

Supplement: Supplementary file 1 — Supplementary Material [file ACM2-15-392-s001.JPG]
